# Supplementary material for: Schnider and Eleveld Models for Propofol Target-Controlled Infusion Anesthesia: A Clinical Comparison
Source: Life (Basel). 2023 Oct 16;13(10):2065. doi: 10.3390/life13102065 (PMC10608783; doi:10.3390/life13102065)
Supplement: Supplementary file 1 [file life-13-02065-s001.zip › life-2628903-supplementary.pdf]

Descriptive analysis of population of female patients undergoing breast surgery

Variables	Total patients<br>n. 78	Total adult patients<br>n. 37	Total older patients<br>n. 41	Total adults vs. elders<br>P-value
Age, yrs	65 [58.2-71]	58 [53-61]	71 [67-74]	<0.001
• Age ≥65 yrs, n (%)	41 (52.6)	0 (0)	41 (100)	<0.001
Weight, kg	65 [58.2-72.7]	64 [58-68]	67 [62-75]	0.114
Height, cm	164 [160-168]	165 [160-170]	164 [160-167]	0.195
BMI, kg/m^2	23.9 [22.3-27.6]	23.2 [21-26.6]	24.9 [23-28]	0.017
• BMI ≥30, n (%)	9 (11.5)	2 (5.4)	7 (17.1)	0.159
ASA, I/II/III, n (%)				<0.001
• I	16 (20.5)	16 (43.2)	0 (0)	
• II	58 (74.4)	20 (54.1)	38 (92.7)	
• III	4 (5.1)	1 (2.7)	3 (7.3)	
Propofol total dose, mg	517.3 [401.4-624.1]	512 [450.6-664.1]	522 [379.6-573.3]	0.344
Surgery time, min	44.5 [34.2-61]	45 [34-81]	44 [36-54]	0.420
Anesthesia time, min	65 [54.2-83.5]	66 [55-107]	65 [54-75]	0.273
**LoR**				
BIS baseline	97 [97-98]	97 [97-98]	97 [96-98]	0.176
CeP LOR, µg/ml	2.62 [1.64-3.66]	2.37 [1.62-3.50]	2.7 [1.7-3.80]	0.462
BIS at LoR	79 [72-83]	79 [65-83]	80 [75-83]	0.469

Time to LoR, min	2 [2-4]	2 [1-4]	3 [2-4]	0.147
**Anesthesia maintenance**				
BIS at $CePMA_1$	48 [44-50]	48 [44-50]	46 [44-52]	0.731
$CePMA_1$, µg/ml	2.70 [2.40-3.20]	2.90 [2.40-3.10]	2.50 [2.40-3.30]	0.581
Time to $CePMA_1$, min	22.5 [19-29.7]	23 [19-30]	22 [19-29]	0.888
BIS at $CePMA_2$	48 [44-50]	48 [44-50]	46 [44-50]	0.863
$CePMA_2$, µg/ml	2.50 [2.30-3.00]	2.70 [2.20-3.00]	2.50 [2.30-2.80]	0.240
**RoR**				
CeP at RoR, µg/ml	0.97 [0.60-1.49]	1.05 [0.73-1.57]	0.77 [0.55-1.42]	0.213
BIS at RoR	73 [66-78]	72 [66-81]	73 [70-78]	0.691
Time to RoR	9 [7-12]	10 [7-12]	9 [7-12]	0.327
Δ CeP, µg/ml	1.72 [0.52-2.87]	1.27 [0.37-2.65]	2.23 [0.70-3.10]	0.098
**Unwanted events**				
DAE, n (%)	39 (50)	21 (56.8)	18 (43.9)	0.365
BSE, n (%)	13 (16.7)	6 (16.2)	7 (17.1)	1.00
LAE, n (%)	3 (3.8)	2 (5.4)	1 (2.4)	0.601
USRE, n (%)	3 (3.8)	2 (5.4)	1 (2.4)	0.601

BMI: body mass index; ASA: American Society of Anesthesiologists physical status classification; LoR: loss of responsiveness; MA: maintenance of anesthesia; RoR: return of responsiveness; BIS: Bispectral Index; CeP: concentrations at the effect site (Ce) of propofol; $CePMA_1$: initial CeP during MA; $CePMA_2$: final CeP during MA; Δ CeP: difference between CeP LoR and CeP ROR; LAE: lightening of anesthesia event; USRE: unwanted spontaneous responsiveness event; DAE: deepening of anesthesia event; BSuppE: burst suppression event.

Figure S1. Dispersion graph of the relation between CeP LoR and age, height, body weight and BMI in female patients undergoing propofol total intravenous anesthesia (TIVA) with target controlled infusion (TCI) using Schnider PK-PD model (39 patients) and Eleveld PK-PD model (39 patients) for breast surgery. Bravais-Pearson's correlation test was used to determine the strength and direction of association between CeP LoR and age, height, body weight, body mass index (BMI). Correlation coefficient (CC), 95% confidence interval (95%CI), and p-values were determined.

Figure S2. Dispersion graph of the relation between BIS-guided CePMA1 and age, height, body weight and BMI in female patients undergoing propofol undergoing propofol total intravenous anesthesia (TIVA) with targeted controlled infusion (TCI) using Schnider PK-PD model (39 patients) and Eleveld PK-PD model (39 patients) for breast surgery. Bravais-Pearson' s correlation test was used to determine the strength and direction of association between BIS-guided initial CeP and age, height, body weight, body mass index (BMI). Correlation coefficient (CC), 95% confidence interval (95%CI), and p-values were determined.

Figure S3. Dispersion graph of the relation between BIS-guided CePMA2 and age, height, body weight and BMI in female patients undergoing propofol undergoing propofol total intravenous anesthesia (TIVA) with target controlled infusion (TCI) using Schnider PK-PD model (39 patients) and Eleveld PK-PD model (39 patients) for breast surgery. Bravais-Pearson' s correlation test was used to determine the strength and direction of association between BIS-guided final CeP and age, height, body weight, body mass index (BMI). Correlation coefficient (CC), 95% confidence interval (95%CI), and p-values were determined.

Figure S4. Dispersion graph of the relation between CeP RoR and age, height, body weight and BMI in female patients undergoing undergoing propofol total intravenous anesthesia (TIVA) with targeted controlled infusion (TCI) using Schnider PK-PD model (39 patients) and Eleveld PK-PD model (39 patients) for breast surgery. Bravais-Pearson' s correlation test was used to determine the strength and direction of association between CeP RoR and age, height, body weight, body mass index (BMI). Correlation coefficient (CC), 95% confidence interval (95%CI), and p-values were determined.

Figure S1

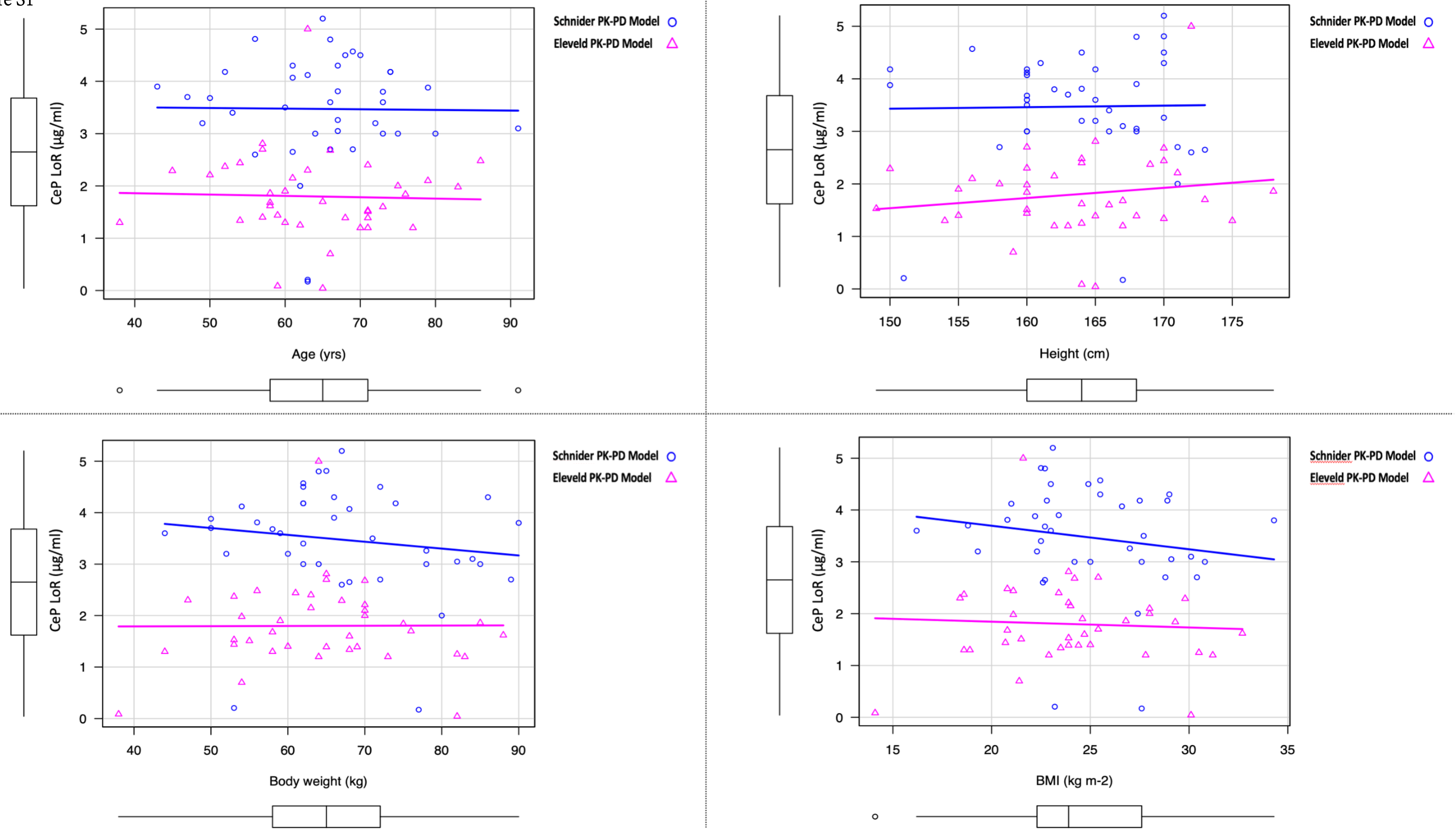

Schnider PK-PD Model
Eleveld PK-PD Model
CeP LoR (µg/ml)
Age (yrs)
Height (cm)
Body weight (kg)
BMI (kg m-2)

Figure S2

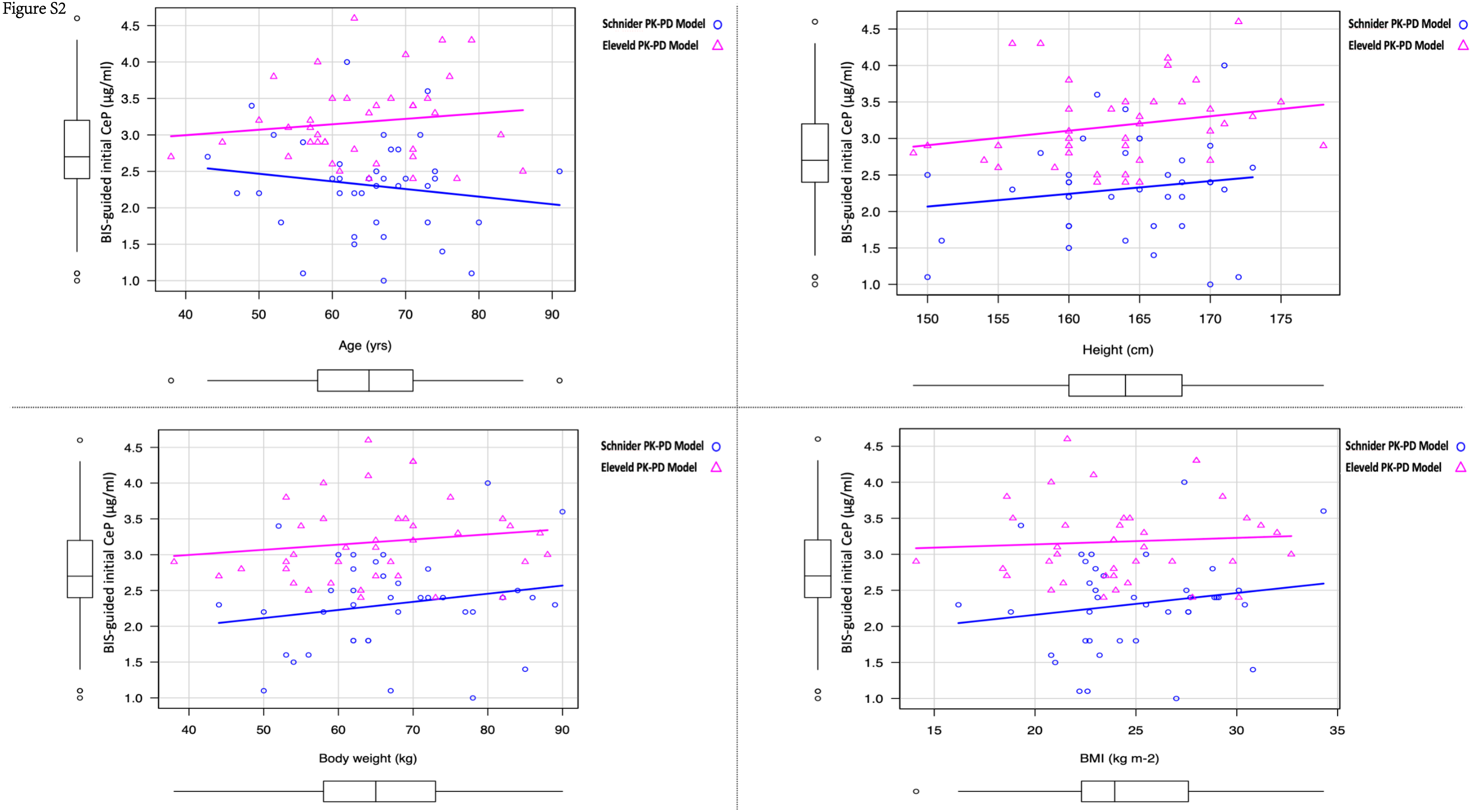

Schnider PK-PD Model
Eleveld PK-PD Model
BIS-guided initial CeP (µg/ml)
Age (yrs)
Height (cm)
Body weight (kg)
BMI (kg m-2)

Figure S3

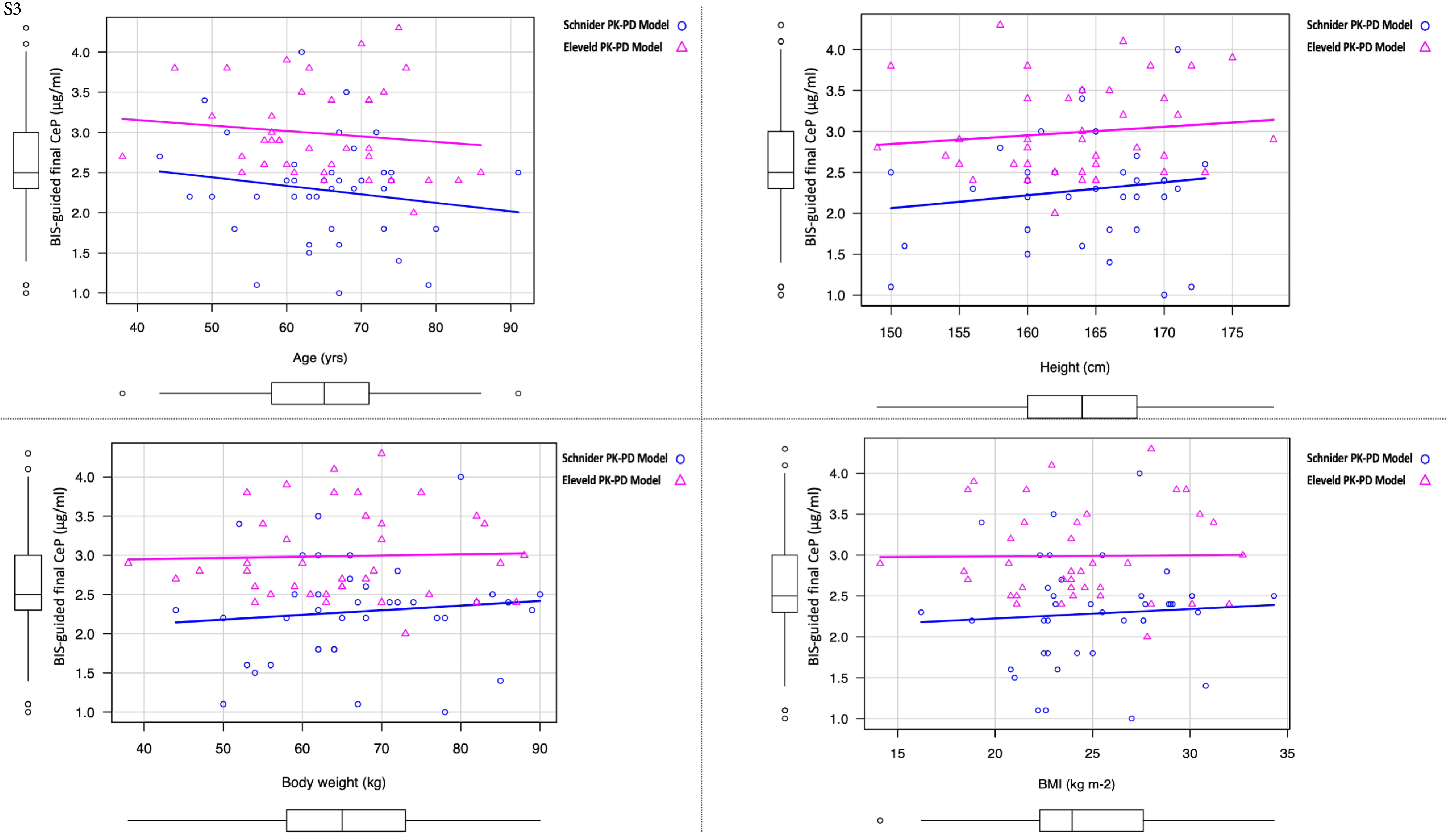
Schnider PK-PD Model
Eleveld PK-PD Model
BIS-guided final CeP (µg/ml)
1.0
1.5
2.0
2.5
3.0
3.5
4.0
40
50
60
70
80
90
Age (yrs)
150
155
160
165
170
175
Height (cm)
Body weight (kg)
15
20
25
30
35
BMI (kg m-2)

Figure S4

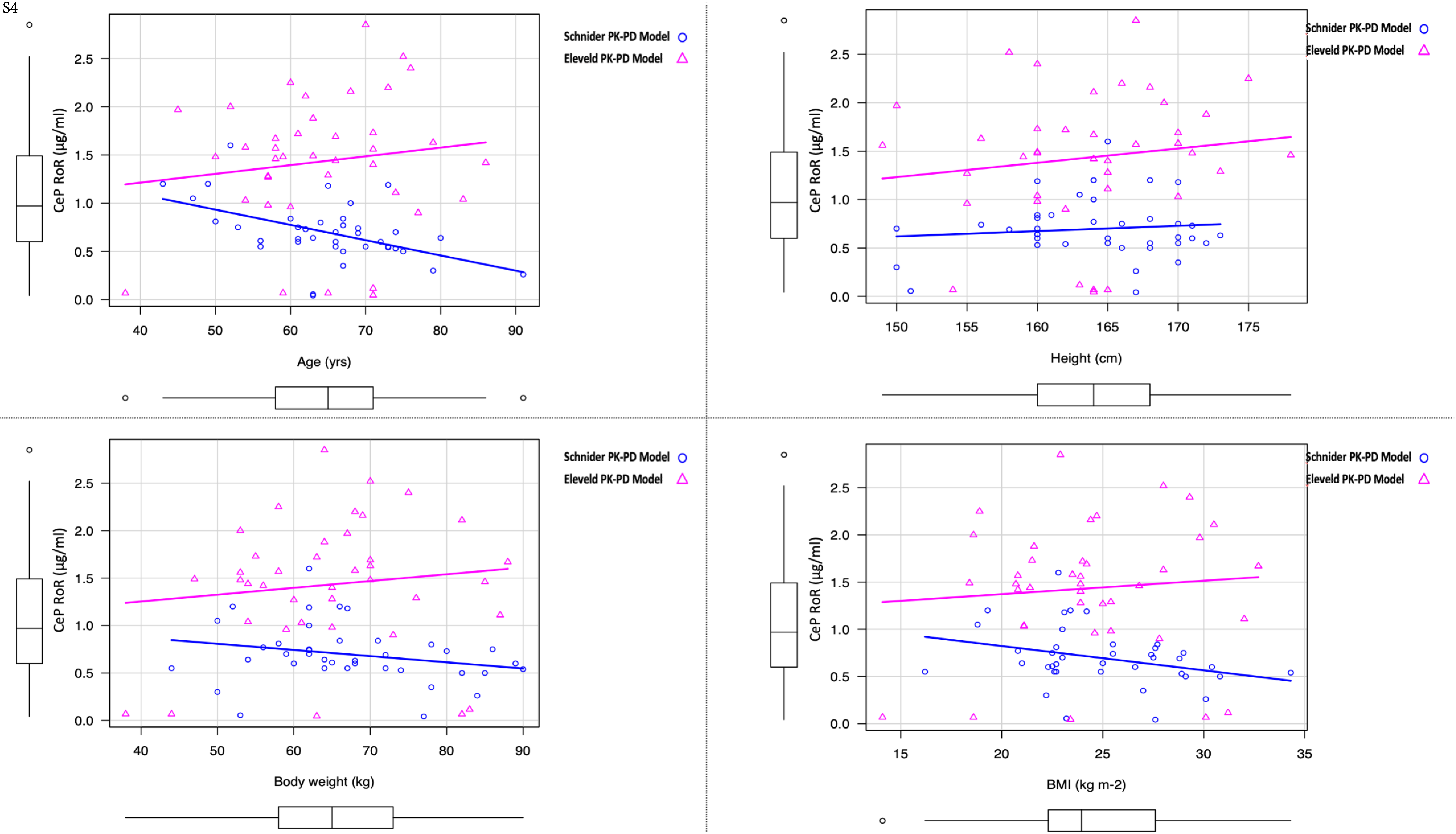
Schnider PK-PD Model
Eleveld PK-PD Model
CeP RoR (µg/ml)
Age (yrs)
Height (cm)
Body weight (kg)
BMI (kg m-2)

Variables		Regression Model					Fitted Regression Model			
**Dependent**	**Independent**	**VIF**	**OR**	**L95%**	**U95%**	***P* value**	**OR**	**L95%**	**U95%**	***P* value**
**DAE**	Age (≥65 years)	**1.408**	0.51	0.15	1.67	0.267				
	Weight (≥65 kg)	**1.690**	1.27	0.28	5.74	0.756	2.70	0.98	7.38	0.053
	Height (≥164 cm)	**1.260**	1.35	0.43	4.20	0.606				
	BMI (≥25 kg/m^2)	**1.805**	1.60	0.408	6.31	0.498				
	ASA physical status (≥2)	**1.446**	1.27	0.28	5.74	0.756				
	Eleveld vs. Schnider Model	**1.091**	5.81	2.05	16.05	<0.001	5.57	2.04	15.20	<0.001
**BSE**	Age (≥65 years)	**1.324**	1.08	0.24	4.70	0.921				
	Weight (≥65 kg)	**1.754**	2.84	0.48	16.7	0.246	2.71	0.71	10.30	0.144
	Height (≥164 cm)	**1.314**	1.05	0.23	4.71	0.946				
	BMI (≥25 kg/m^2)	**1.823**	0.89	0.15	5.06	0.896				
	ASA physical status (≥2)	**1.345**	0.98	0.13	7.32	0.990				
	Eleveld vs. Schnider Model	**1.019**	7.62	1.52	38.30	0.013	7.66	1.54	38.10	0.012

**LAE = USRE**	Age (≥65 years)	**1.389**	0.60	0.03	10.90	0.730				
	Weight (≥65 kg)	**1.811**	0.28	0.01	7.92	0.462				
	Height (≥164 cm)	**1.136**	2.12	0.15	29.40	0.575				
	BMI (≥25 kg/m^2)	**2.011**	2.32	0.07	76.08	0.637				
	ASA physical status (≥2)	**1.502**	0.58	0.02	12.60	0.734				
	Eleveld vs. Schnider Model	**1.065**	2.13	0.16	27.00	0.560				

Notes. Logistic regression analysis was performed to explain the relationship between one dependent variable and various independent variables. Categorical independent variables were dichotomized as follows: age (younger patients [18–64 years] vs. older patients [≥65 years]); BMI (normal weight [18.5–24.9 kg/m^2] vs. overweight [BMI ≥25 kg/m^2]); ASA physical status (ASA 1 vs. ASA 2-3); weight and height were dichotomized according to mean value for continuous normally distributed variables or median value for continuous non-normally distributed variables. Odds Ratios with 95% confidence intervals were determined. Multicollinearity was assessed using variance inflation factors. Akaike information criterion and backward/forward stepwise regression analysis were used to choose the best model.

Abbreviations. BMI: body mass index; VIF: variance inflation factor; OR: Odds Ratio; L95%: lower limit of the 95% CI; U95%: upper limit of the 95% CI. ASA: American Society of Anesthesiologists; BMI: body mass index; LAE: lightening of anesthesia event; USRE: unwanted spontaneous responsiveness event; DAE: deepening of anesthesia event; BSuppE: burst suppression event.
